# Editorial: Cognitive Disorders in Neuroimmunological Diseases

**DOI:** 10.3389/fneur.2020.00169

**Published:** 2020-03-24

**Authors:** Tjalf Ziemssen, Dawn Wendy Langdon, Pasquale Calabrese

**Affiliations:** ^1^MS Center, Center of Clinical Neuroscience, Department of Neurology, University Clinic Carl-Gustav Carus, Dresden University of Technology, Dresden, Germany; ^2^Royal Holloway University of London, Egham, United Kingdom; ^3^Neuropsychology and Behavioral Neurology Unit, Division of Molecular and Cognitive Neuroscience, University of Basel, Basel, Switzerland; ^4^Department of Neurology, University Clinic of Basel, Basel, Switzerland

**Keywords:** multiple sclerosis, cognition, neuromyelitis optic (NMO), limbic encephalitis, neuroimmunology

In recent decades there has been a rapid growth in the discipline of neuroimmunology: A new area of research investigating how the immune system interacts with the brain, and affects brain function, for example with cognition (1). A convergence of multidisciplinary investigators helped to launch this fascinating research field by developing several groundbreaking lines of research. The close connection between the nervous, endocrine, and immune systems has become a major challenge for interdisciplinary research. Newly developed methods in neuropsychology, immunology, and imaging allow deeper insights into the mechanisms of neuroimmune interactions (2, 3).

In this Research Topic, the role of neuroinflammation on cognition and neuropsychology, basics of psychoneuroimmunology and neuropsychology including conditioned immunomodulation as well as diagnostic aspects such as measurement of cognition are presented (4). In addition, case reports can demonstrate cognitive problems, emotional disturbance, behavioral abnormalities and psychiatric symptoms beyond reviews and original papers. Beyond multiple sclerosis (MS), other neuroimmunological diseases as neuromyelitis optica (NMO) or limbic encephalitis are presented in this Research Topic.

In their review, Brochet and Ruet focus on the cognitive impairment in Multiple Sclerosis (MS) with regard to disease duration and clinical phenotypes. Whereas, the effect of disease duration seems to be confounded by the effect of age, the MS phenotype itself seems to play an important role in the cognitive profile of patients because of its specific pathological mechanism.

To characterize cognitive impairment in MS in a more detailed way, Yalachkov et al. demonstrate that MS patients exhibit an altered multisensory perception to sound-induced flash illusion, a multisensory perceptual illusion, and that their susceptibility to the perceptual illusion is negatively correlated with their neuropsychological test performance. This paradigm may serve as a screening test for cognitive deficits in MS patients.

Moving from MS to NMO spectrum disorders (NMOSD), Oertel et al. review the highly prevalent, but often unrecognized and insufficiently treated, cognitive burden in NMOSD patients; this consists mainly of problems in attention and memory and may reflect a clinical correlate changes which are independent of attacks.

Brain imaging can help to study the link between cognitive dysfunction and alterations of white matter connectivity using graph theory, as Bin Cho et al. report, in 14 NMO patients vs. 21 healthy controls. Overall white matter disruption and sub-network alterations as manifestation of network dysconnectivity seem to be crucial in pathophysiology of cognitive impairment in NMO.

Beyond imaging, biomarkers could be of interest as Qiao et al. have investigated in patients with primary Sjögren's syndrome with and without NMOSD. Serological comparative proteomics including serum clusterin and complement factor H demonstrated significant differences between these entities.

Moving to limbic encephalitis, Hansen discusses long-term memory dysfunction in these patients: in translational transfer experiments of autoimmune encephalitis from humans to the mouse a critical impairment of synaptic long-term potentials in the hippocampus was induced by autoantibodies against the N-methyl-D-aspartate receptor (NMDAR) and alpha-amino-3-hydroxy-5-methyl-4-isoxazolepropionic acid receptor subunit GluA2 which might represent a potential cellular mechanism of mnestic dysfunction.

In line with this opinion, Wang et al. present in their original research that a decrease in amplitude of the NMDAR-mediated excitatory postsynaptic currents in the hippocampal neurons could be shown in rats treated with anti-NMDAR antibodies clinically linked to an impaired learning performance. Both papers suggest a significant role of neuroinflammation in exacerbating the memory impairment in anti-NMDAR disease which could be a potential target to delay or reverse memory impairment.

An autoimmune encephalitis could be mediated by antibodies to Metabotropic Glutamate Receptor 5 (mGluR5) Antibodies in Cerebrospinal Fluid, as Guevara et al. describe in a case report. This complex case tells us that antiinflammatory treatment should not be delayed for the antibody results when there is a reasonable index of suspicion of autoimmune encephalitis.

This is especially true as the exact target antigen of autoantibodies may not yet be known: Thanarajah et al. present an atypical case of autoimmune encephalitis with cerebellar and temporal dysfunction but without seizures associated with high levels of cerebrospinal fluid neuropil antibodies against a yet unknown epitope on the neuronal surface in the cerebellum, hippocampus, thalamus, and the olfactory bulb.

## Author Contributions

All authors listed have made a substantial, direct and intellectual contribution to the work, and approved it for publication.

### Conflict of Interest

TZ has received reimbursements for participation in scientific advisory boards from Almirall, Bayer, Biogen, Celgene, Novartis, Merck, Sanofi, Teva. He has also received speaker honorarium from Bayer, Biogen, Celgene, Genzyme, Novartis, Teva, Sanofi. He has also received research support from Biogen, Novartis, Sanofi, Teva. DL has participated in speaker bureau for Bayer, Merck, Almirall, Excemed, TEVA, Roche, Novartis, Biogen, Sanofi; has had consultancy from Novartis, Bayer, Merck, Biogen, TEVA, Sanofi; has had research grants from Bayer, Merck, Novartis, Biogen. PC has received honoraria for speaking at scientific meetings, serving at scientific advisory boards and consulting activities from: Abbvie, Actelion, Almirall, Bayer-Schering, Biogen Idec,Celgene, EISAI, Genzyme, Lundbeck, Merck Serono, Novartis, Pfizer, Teva, and Sanofi-Aventis. His research is also supported by the Swiss Multiple Sclerosis Society and the Swiss National Research Foundation.
